# Education and Research during Pandemics: Illustrated by the Example of Experimental Biocomposites Research

**DOI:** 10.3390/polym12081848

**Published:** 2020-08-18

**Authors:** Oisik Das, Seeram Ramakrishna

**Affiliations:** 1 Department of Engineering Sciences and Mathematics, Luleå University of Technology, 97187 Luleå, Sweden; 2 Department of Mechanical Engineering, National University of Singapore, Singapore 117575, Singapore

In late 2019, a novel Coronavirus was detected in Wuhan city of China, giving rise to the catastrophic pandemic that is still rampant today. Initially, the worst-hit districts were put under lockdown, which then extended to cities and eventually whole countries. Travel of people, along with logistics of goods and services, were (and still are) severely affected. Most nations of the world urged their citizens to stay indoors so as to avoid exposure to the virus, and thus remain infection-free. One of the demographics that are negatively affected by the lockdown measures is the students and researchers. Numerous universities around the world had to shut their premises at short notice, thus prompting a rapid shift from in-classroom education to online education, a transition that normally would take decades to happen. In particular, students received their classes through digital platforms, which included Zoom, Microsoft Teams, Skype, etc., whereas the researchers adopted tele-working. Although this strategy employed by universities is effective in curbing the further spread of the virus, it has some unintended consequences. Firstly, owing to the uncertainly regarding the end date for the current coronavirus pandemic, millennials and freshmen are unsure about their immediate enrolment in their chosen courses and programmes. For example, the University of Ohio in USA and the University of Cambridge in the United Kingdom will hold online classes for the upcoming fall and until the summer of 2021, respectively. This is particularly disheartening for international students, who are anticipating an active academic experience that includes campus life, engagement in classrooms, obtaining in-person feedback from lecturers, bonding and networking in cafes, etc. Secondly, and more importantly, students whose programmes warrant undertaking a significant amount of laboratory work are stressed about the stagnant nature of their research. While a few fields of study can be conducted on a digital platform, experimental research requires the presence of the person in laboratories for a substantial amount of time. Biocomposite education is at its core an experimental one, which includes the design of the biocomposite, preparation of raw materials, fabrication and manufacturing, prototyping, and finally testing and characterisation. Therefore, it is critical to identify some effective means to propagate biocomposites education during pandemics, wherein students and researchers are confined to quarantines. In other words, educators should create paths for effective learning in the biocomposite field in a distanced education system via alternative routes and remote controlled laboratories and equipment.

In light of the aforementioned, five strategies could be adopted by the students and researchers to sustain biocomposites education and learning during viral outbreaks and disruptions. The first strategy, which is one of the most obvious ones, is to bolster the theoretical knowledge regarding composite science and technology. Often, a student or a researcher learns on the job, i.e., learning by doing. While this is imperative to activate the psychomotor taxonomic domain, the cognitive domain can be made robust by indulging in the comprehension of background knowledge regarding various scientific phenomena and engineering concepts [1]. Although a student can progress through his/her academic career and reach higher positions of lecturer or assistant professor by relying solely on the ‘working knowledge’ of biocomposites, an in-depth understanding of concepts like micromechanics, macromechanics, laminate theory, structural mechanics, analytical modelling and finite element modelling will make them reflective practitioners [2]. Additionally, these academics will be intrinsically motivated [3] to conduct effective teaching and ground-breaking research. Therefore, the imparting of theoretical knowledge on biocomposites will garner self-regulation [4], confidence and self-efficacy [5] in the students and researchers.

In the second strategy, the students and researchers can devote their time to preparing comprehensive and critical review articles meant for beginners and experienced researchers, respectively. Not only does the preparation of review articles inadvertently facilitate the absorbance of overall knowledge, but also their eventual publication in peer-reviewed journals attracts more citations (compared to the narrowly focused research articles), which will boost the person’s academic career and visibility. The writing of review articles enables the author to develop a holistic overview regarding specific aspects of the biocomposite field. Additionally, the author becomes aware of the latest developments in the state-of-the-art research, and is able to critically analyse and well position his/her own research so as to address specific scientific and technological challenges and needs. Thus, the above-mentioned facets of writing a review article are conducive for the development of biocomposites education because students/researchers will learn by immersing themselves in loops of experience, theories and practice, as specified by Boyatzis and Kolb, 1995 [6]. 

In the third strategy, the students and researchers can perform life cycle analyses (LCA) of various biocomposite products. LCA does not require access to laboratories, and thus can be performed from the safety of one’s home. Through LCA analysis, the student/researcher will be able to grasp the importance of manufacturing and environmental sustainability, and attaining a circular economy mind-set. It is critical to reduce greenhouse gas (GHG) emissions and wastage at every stage of the biocomposites’ life cycle, and LCA will shine light into the environmental impact of sourcing raw materials and feedstock, processing, manufacture, distribution, use, repair, maintenance and disposal or recycling, i.e., the cradle-to-grave life of the product. The performing of LCA studies will not only create opportunities for journal publications, but also encourage the student/researcher to undertake industry-facing and market-oriented sustainable design and re-design of biocomposites in the future. This will lead to the academic being environmentally conscious and striving towards waste minimisation and pollution reduction during the biocomposite’s development and life cycle. 

The fourth strategy is related to simulation studies of various aspects of biocomposites. Simulation studies can be related to the determination of process feasibility parameters, its lifetime prediction, failure mechanisms, etc. Although simulation without experimental validation could be futile, students/researchers can delve into the modelling world, which can enable process optimisation and effective product life cycle engineering. Furthermore, the students/researchers can visualise the performance of the biocomposite without having to actually manufacture the product. Therefore, simulation studies will not only enhance one’s theoretical understanding of composite science, but also prepare one to tailor the design in order to have desirable performance properties and functionalities. Simulation studies will be the closest thing for the students/researchers to experimentally designing and developing biocomposites, and characterising their various properties in a manner akin to a real-life laboratory session. 

If performing real-world experiments is unavoidable, maybe the students/researchers can do so in a simulated laboratory environment of virtual reality (VR), which is the fifth strategy. Nevertheless, VR technology would not be accessible to all the students, especially in developing nations where such technologies could be non-existent. VR technology can potentially allow students/researchers to collaborate and interact with the artificially created biocomposite laboratory by moving through its spaces and experiencing visual and auditory feedback from common instruments, such as injection moulding machines, Instron Universal testing machines, cone calorimetry equipment, etc. Since VR has been used in medicine in a way that has allowed the trainee doctors to rectify errors [7], the same can be emulated in biocomposite education. VR in biocomposite education will be beneficial in enabling the student/researcher to develop his/her experimental skills, and will reduce the total cost of the programme, since raw materials will not be expended. 

In summary, there are several ways by which a student or a researcher can be immersed in continuing biocomposites education during pandemics and massive disruptions. Adherence to the aforementioned strategies will ensure that students/researchers can come back with a strong foundation once the pandemic ends and the laboratories reopen. The following Figure 1 depicts the ideas put forward in this article. An ideal solution for maintaining the flow of biocomposites research and education is the combination of all the five strategies in some form or another. 

## Figures and Tables

**Figure 1:**
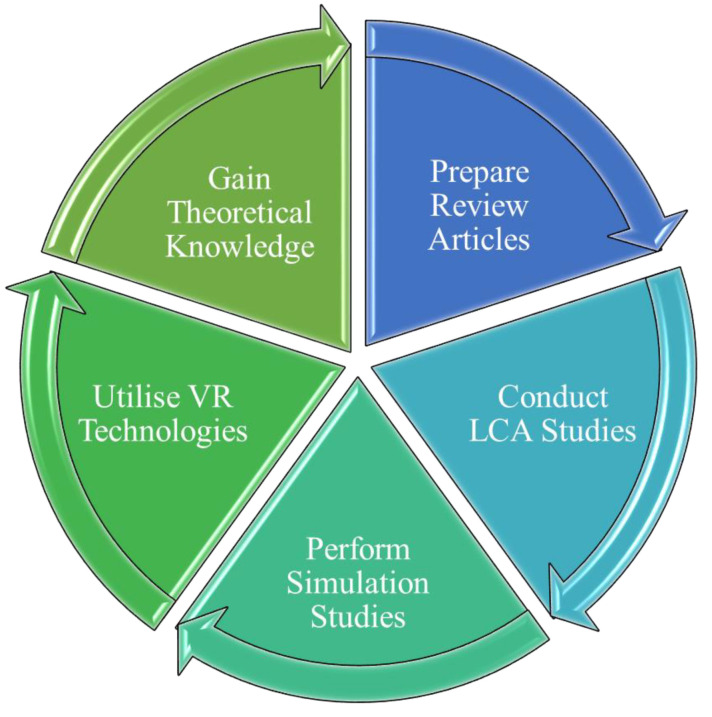
The five strategies for students and researchers to adopt in order to maintain the continuity of biocomposites education during a pandemic.

## References

[1] Adesoji F.A. (2018). Bloom taxonomy of educational objectives and the modification of cognitive levels. Adv. Soc. Sci..

[2] Schön D.A. (1987). Educating the Reflective Practitioner.

[3] Rust C. (2002). The impact of assessment on student learning: How can the research literature practically help to inform the development of departmental assessment strategies and learner-centred assessment practices?. Act. Learn. High. Educ..

[4] Ng E.M. (2018). Integrating self-regulation principles with flipped classroom pedagogy for first year university students. Comput. Educ..

[5] Baker D. (2013). What works: Using curriculum and pedagogy to increase girls’ interest and participation in science. Theory Pract..

[6] Boyatzis R.E., Kolb D.A. (1995). From learning styles to learning skills: The executive skills profile. J. Manag Psychol..

[7] Li L., Yu F., Shi D., Shi J., Tian Z., Yang J., Wang X., Jiang Q. (2017). Application of virtual reality technology in clinical medicine. Am. J. Transl..

